# Myeloperoxidase as a Potential Biomarker of Acute-Myocardial-Infarction-Induced Depression and Suppression of the Innate Immune System

**DOI:** 10.3390/antiox11112083

**Published:** 2022-10-22

**Authors:** Andreas Baranyi, Dietmar Enko, Andreas Meinitzer, Dirk Von Lewinski, Hans-Bernd Rothenhäusler, Leonhard Harpf, Heimo Traninger, Barbara Obermayer-Pietsch, Birgit M. Harb, Melanie Schweinzer, Moritz Platzer, Sieglinde Zelzer

**Affiliations:** 1 Division of Psychiatry and Psychotherapeutic Medicine, Medical University of Graz, 8036 Graz, Austria; 2 Clinical Institute of Medical and Chemical Laboratory Diagnostics, Medical University of Graz, 8036 Graz, Austria; 3 Division of Cardiology, Department of Internal Medicine, Medical University of Graz, 8036 Graz, Austria; 4 PRO-DOC Cardiac Rehabilitation, 8020 Graz, Austria; 5 PRO-REHA Cardiac Rehabilitation, 8020 Graz, Austria; 6 Endocrinology Lab Platform, Division of Endocrinology and Diabetology, Department of Internal Medicine, Medical University of Graz, 8036 Graz, Austria; 7 SKA-RZ St. Radegund für Herz-Kreislauferkrankungen, 8061 St. Radegund bei Graz, Austria; 8 Klinikum Favoriten, 1100 Vienna, Austria

**Keywords:** myeloperoxidase, depression, innate immune system, acute myocardial infarction

## Abstract

While myeloperoxidase (MPO) serves as an indicator of both neutrophil and innate-immune-system function, the potential suppression of the innate immune system in patients with acute myocardial infarction (AMI)-induced depression might be evidenced by a decrease in MPO serum levels. The aim of this prospective study was to (1) determine whether serum concentrations of MPO vary immediately and 6 months after AMI and (2) to investigate whether MPO concentrations at the time of the AMI are significant predictors of AMI-induced depression and the depression-associated suppression of the innate immune system. A total of 109 AMI patients were assessed with the Hamilton Depression Scale (HAMD-17) immediately after admission to the hospital and 6 months later. The MPO status was assessed with serum samples, which were also collected immediately and 6 months after AMI. The depressive patients showed significantly lower MPO blood levels immediately and 6 months after the AMI compared to the patients without depression (ANCOVA: MPO (depression) F = 4.764, df = 1, *p* = 0.031). The baseline MPO was observed as a significant predictor (*p* = 0.027) of AMI-induced depression 6 months after AMI. MPO is a potential biomarker for AMI-induced depression, indicating a depression-associated suppression of the innate immune system.

## 1. Introduction

### 1.1. Depression and Myocardial Infarction

Depression is about three times more common in patients after acute myocardial infarction (AMI) than in the general community [1,2]. Almost half of patients recovering from AMI show symptoms of depression and 15% to 20% even suffer from major depression [3]. AMI is followed by a range of stress responses, such as the activation of proinflammatory cytokines, of the sympathetic nervous system and the hypothalamic–pituitary–adrenal (HPA) axis. In some cases, the dysregulation of these systems persists and develops into depression [4,5,6]. 

### 1.2. Depression, Myocardial Infarction and Inflammation

Proinflammatory cytokines are secreted by the cells of the innate and adaptive immune system in response to an antigen and act as chemical messengers for regulating the innate and adaptive immune systems. These pro-inflammatory cytokines are elevated in many patients with cardiovascular diseases and predict the risk of cardiovascular events, including coronary events, in the future [7,8,9]. However, immune responses can be triggered not only by exposure to the components of bacteria and viruses, such as llipopolysacharides (LPS) and synthetic dsRNA, but also by severe physiological and psychological stress, such as AMI. As a result, signaling-transduction cascades are activated and proinflammatory cytokines, such as interleukin-6 (IL-6), tumor necrosis factor-alpha (TNF-α), interferon (IFN), chemokines and acute-phase proteins, are produced [10]. 

There are distinct associations between proinflammatory cytokine elevations and subsequent alterations in mental state. Thus, several studies have shown that proinflammatory cytokines (e.g., IL-6, TNF-α), as well as LPS, often cause sickness behavior, even in mentally healthy patients with infectious diseases. Sickness behavior is a multifaceted symptom complex that comprises lethargy, apathy, low mood, anxiety, sleep disturbances, lack of appetite and social retreat. This sickness behavior in otherwise mentally healthy patients is very similar to the symptoms of psychiatric patients with depressive episodes [11].

These similarities with the symptoms of depression and, in particular, previous research on INF-α gave the impetus to the inflammatory hypothesis of depression, also called the cytokine hypothesis of depression [12]. Thus, IFN-α, TNF-α and IFN-γ activate the enzyme indoleamine 2,3-dioxygenase (IDO) [12,13]. As a consequence, the activated IDO leads to an increased degradation of the serotonin precursor, tryptophan, to kynurenine and, subsequently, to neurotoxic kynurenine metabolites (primarily quinolinic acid). Quinolinic acid acts as an agonist in the glutamatergic *N*-methyl-D-aspartate receptor and causes the overactivation of this receptor [13,14,15,16]. In the last few years, numerous studies further revealed that besides IFN-α, the proinflammatory cytokines IL-6 and TNF-α and increased blood levels of the C-reactive protein (CRP), in particular, are associated with depressive disorders.

In summary, inflammation might be closely related to both depression and cardiovascular diseases [9]. For example, CRP is a marker of inflammation and predicts depression and cardiovascular risk [17]. Another well-known proinflammatory cytokine associated with both disease entities is IL-6 [8,18]. Wilkowska et al. [19] reported that a strong inflammatory response as an element of the stress reaction after AMI might predispose individuals to subsequent depression. In the same study, IL-6, TNF-α, IL-17a and IL-12p70 were elevated in the depression group on the third day after AMI compared to the control group. On the fifth day, IL-6 and IL-17 were still elevated. Therefore, the risk of developing post-AMI depression might be significantly higher in patients with a stronger inflammatory response, as expressed by higher pro-inflammatory-cytokine blood levels during the first days after AMI [19]. The neuroinflammation can persist for a long time after the initial peripheral inflammation has subsided. [20]. In addition, Shang et al. [21] reported that the presence of depressive symptoms is positively associated with TNF-α blood levels among patients who have suffered from AMI.

### 1.3. Links of Proinflammatory Cytokines and the Hyperactivity of the HPA Axis

AMI-related physiological and psychological stress is associated with neuroinflammation (increased levels of proinflammatory cytokines), the activation of the HPA axis, and the subsequent release of the glucocorticoid, cortisol [11,22,23,24]. Cortisol is able to decrease physiological- and psychological-stress-related increased pro-inflammatory cytokine levels. Subsequently, glucocorticoid receptors in the hippocampus detect these stress-related increased cortisol levels. As a consequence, the hippocampus regulates the hypothalamus via a negative-feedback loop to decrease the corticotropin-releasing hormone and, subsequently, the blood concentrations of cortisol [25]. 

However, glucocorticoid-receptor resistance, hyperactivity of the HPA axis, and hypercortisolism are well-known neurobiological changes in depressive patients. Furthermore, a manifest dysfunction of the HPA axis is presented in about 70% of patients with depressive disorders [6,26]. In a study by Wilkowska et al. [5], patients with depressive symptoms after an AMI had a flattened diurnal-serum-cortisol profile.

### 1.4. Depression and the Innate Immune System

Major depression is often associated with systemic immune activation. However, there is some evidence that major depression might also be associated with immunosuppression. Thus, deficits of the innate immune system in people with major depression have been suspected and deficits in the function of the NK cells have, in particular, been linked to the development of depressive episodes [11]. A study by Duggal et al. [27] also revealed the possible suppression of the innate immune system by psychological stress and depressive disorders. In this study, elderly patients with hip fractures were examined. The results of this study showed that the stress load led to depressive episodes, to a reduction in neutrophil function, and to poorer neutrophil-superoxide production.

Studies investigating the immunological and endocrinological profiles of patients with depression after AMI might provide valuable insights into the pathophysiology of this patient setting [5].

### 1.5. Myeloperoxidase (MPO) as a Biomaker of the Activity of the Innate System

Neutrophil granulocytes have a variety of oxidative and non-oxidative mechanisms for the effective killing of bacteria [28,29,30]. A key part of the oxidative immune-defense mechanisms of neutrophil granulocytes is MPO, a peroxidase enzyme, which produces the strong oxidant, hypochlorous acid, from chloride and hydrogen peroxide [31]. These MPO–hydrogen-peroxide-halide systems are effective at neutralizing a wide range of microorganisms [30,31,32,33,34]. Therefore, MPO concentrations allow the estimation of the activity of neutrophil granulocytes and the suppression of innate immunity.

## 2. Objectives

After AMI, many patients suffer from severe and prolonged stress [35], which is associated with neuroinflammation and vulnerability to depression. In particular, IL-6 plays an important role in the inflammatory hypothesis of depression. However, there is also some evidence that major depression might be associated with immunosuppression [11]. Since MPO serves as an indicator of neutrophil function and the function of the innate immune system, the potential suppression of the innate immune system in patients with AMI-induced depression might be evidenced by a decrease in MPO blood levels.

Therefore, the aim of this first prospective study was to (1) explore whether IL-6 levels are associated with depression, (2) investigate whether MPO serum concentrations vary immediately and 6 months after AMI and (3) to investigate whether MPO is a significant predictor of AMI-induced depression, indicating the depression-associated suppression of the innate immune system.

## 3. Methods and Materials

### 3.1. Methods

The study was performed at the Division of Cardiology, Department of Internal Medicine, Medical University of Graz, 8036 Graz, Austria. In total, 111 AMI patients were included in the study; however, of these 111 study participants, two died of complications of AMI shortly after hospitalization. The following exclusion criteria for enrollment in this study were defined: (1) pre-existing delirium, (2) dementia, (3) depression and other mental illnesses present at the time of the AMI and (4) any additional acute and severe internal disease other than AMI at the time of AMI diagnosis.

Shortly after admission to the hospital and 6 months after AMI, all participants were assessed with a clinical psychiatric interview based on the Hamilton Depression Scale (HAMD-17) [36,37]. None of the 111 participating AMI patients had clinically significant depression at the time of hospital admission.

Serum samples for determination of MPO and IL-6 were collected at the time of hospitalization and 6 months after AMI, respectively.

This study was approved by the Institutional Review Board of the Medical University of Graz (approval number: 28-126 ex 15/16). Prior to study enrollment, all study participants were required to sign an informed consent form. 

### 3.2. Diagnosis of AMI

All patients underwent percutaneous coronary intervention (PCI). AMI was defined with at least one troponin T value above the 99th percentile and new ischemic changes on the electrocardiogram (ECG). In addition, a successful PCI of the culprit lesion was required.

### 3.3. Psychiatric Diagnoses

Hamilton rating scale for depression (HAMD-17,Hamilton et al. [37]): By applying the observer-rating scale HAMD-17, the severity of depressive symptoms was determined immediately after AMI and 6 months later. The severity of depression was classified as (1) no depression (HAMD-17 total score of 0–7]) (2) mild depression (HAMD-17 total score of 8–16), (3) moderate depression (HAMD-17 total score of 17–23), and (4) severe depression (HAMD-17 total score of ≥24) [36].

Immediately after the AMI, pre-existing psychiatric diagnoses other than depression were queried in an ICD-10-based clinical psychiatric interview. 

### 3.4. Sociodemographic Characteristics

A sociodemographic questionnaire was used to obtain the participants’ sociodemographic characteristics, age and sex.

### 3.5. Clinical Characteristics of the AMI Patients

The following AMI-related clinical characteristics were recorded:

- Cardiac status at the time of AMI: type of AMI (STEMI/NSTEMI), Killip class stratification (=mortality risk after AMI).
- Severity of myocardial infarction: Troponin T, creatine kinase MB (CK-MB), maximum creatinine kinase (CK max) within the first three days after AMI. CK values were measured at least once daily within the first 3 days after AMI.
- Percutaneous coronary intervention (PCI): coronary artery disease, coronary flow before PCI (TIMI Score), coronary flow after PCI (TIMI Score), as well as single=versus-multivessel PCI.
- In-hospital outcome: AMI-related death, reinfarction, severe bleeding and left-ventricular rejection fraction (LVR %).
- Cardiological risk factors at the time of the AMI: body-mass index (BMI); insulin-dependent diabetes mellitus, arterial hypertension and hyperlipidemia.
- Pre-existing psychiatric morbidity; addictions.

### 3.6. Laboratory Analyses

Interleukin-6 (IL-6) values were measured with a high-sensitivity ELISA kit (DI-ACLONE, Besançon, France). Briefly, a capture antibody highly specific for IL-6 was coated to the wells of a microtiter-strip plate. IL-6 from the samples captured these antibodies and, subsequently, the biotinylated anti-IL-6 secondary bind of this complex. Together with a horse-rapid-peroxidase and a chromogen substrate, a blue-colored complex was developed and the reaction was then stopped by the addition of acid, turning the resulting final product yellow. The intensity of the produced colored complex was directly proportional to the concentration of IL-6 present in the samples and standards. The calculated overall coefficient of variation was 4.4% (range 1.4–11.0%). The limit of detection was <2 pg/mL. 

For this study, serum MPO concentrations were analyzed using the MPO enzyme-linked immunosorbent assay (ELISA) kit (cat. no.: K 6631B; Immundiagnostik AG, Bensheim, Germany). Briefly, serum samples were diluted 1:40 with sample-dilution puffer. A rabbit anti-MPO peroxidase-labeled antibody was used for detection prior to the addition of tetra-methyl-benzidine as a substrate. An acidic stop solution was added to terminate the reaction and the MPO concentration was measured at 450 nm with a VICTOR multilabel plate reader (Perkin Elmer, Waltham, Massachusetts, United States). The concentrations were calculated from a standard curve. The intraassay coefficients of variation (CVs) for MPO were 6.2% (3 μg/L), 7.6% (15 μg/L) and 12.1% (30 μg/L) and the inter-assay CVs were 5.7% (3μg/L), 7.3% (15 μg/L) and 9.1% (30 μg/L). The limit of detection was 1.6 μg/L [38].

### 3.7. Statistical Analyses

AMI-related clinical data are reported as means and standard deviations (SD), or medians and IQR. For analysis of continuous variables, t-tests were used and for categorical variables, χ²-tests or Fisher-exact tests were performed. In the case of multiple comparisons, a Bonferroni-alpha adjustment was performed.

MPO levels in patients with/without AMI-induced depression were analyzed with the analysis of covariance (ANCOVA), in which age, sex, coronary artery disease (number of affected vessels), maximum creatinine kinase (CK max) and BMI were included as covariates. 

Logistic regression models were used to examine the impact of the MPO blood level at the time of hospital admission as a predictor of AMI-induced depression six months after AMI. In the first step of the logistic-regression analyses, MPO levels at the time of hospital admission were regressed against AMI-induced depression six months after AMI (depression six months after AMI: yes/no) (Model 1). In the second step, the common control variables, age, sex and coronary artery disease (number of vessels affected) were introduced into the hierarchical logistic regression model (model 2).

All of the statistical analyses were carried out using SPSS 25.0 for Windows (SPSS; Chicago, IL, USA).

## 4. Results

### 4.1. Sociodemographic Characteristics

The overall sample consisted of 109 AMI patients (90 males, 82.6%; 19 females, 17.4%). All 109 study participants were Caucasian, with a mean age of 60.3 (± 11.3) years.

### 4.2. Depression

**Figure 1:**
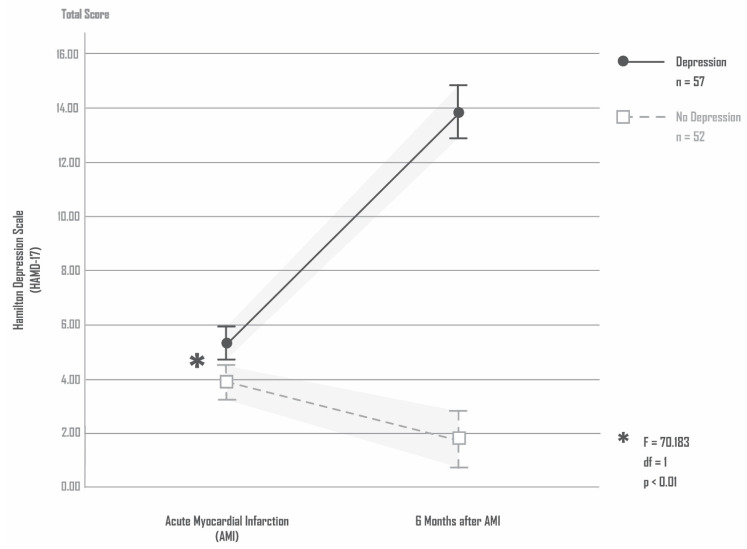
Total HAMD-17 scores for patients with/and without depression 6 months after AMI.

At the time of admission due to an AMI, no patient had clinically significant depression. Overall, 52/109 (47.7%) patients remained free of depression 6 months after AMI, while 57/109 (52.3%) showed depressive symptoms 6 months after AMI. Compared with the time of admission due to an AMI, the patients with subsequent AMI-induced depression had significantly higher total HAMD-17 scores 6 months after the AMI. The patients without subsequent AMI-induced depression had a decrease in total HAMD-17 scores ANOVA: HAMD-17 total score (time) F = 70.183, df = 1, *p* < 0.01; total HAMD-17 score (depression) F = 192.125, df = 1, *p* < 0.01; total HAMD-17 score (depression × time) F = 217.822, df = 1, *p* < 0.01. Figure 1 shows the total HAMD-17 scores for patients with/and without depression 6 months after AMI.

Of all 57 depressive patients, 42 (73.7%) had mild depression (total HAMD-17 score of 8–16); 11/57 (19.3%) had moderate depression (total HAMD-17 score of 17–23); and 4/57 (7%) had severe depression (total score of ≥24 on the HAMD-17).

Regarding sociodemographic characteristics, the patients with depressive symptoms after AMI were more often female (χ² = 6.553, df = 1, *p* = 0.010). No differences between depressive and non-depressive AMI patients were observed with regards to the type of AMI, Killip class stratification, severity of AMI, cardiac risk factors, in-hospital outcomes, or previous mental illness. The depressive and non-depressive AMI patients differed with regards to their coronary flow after PCI, as measured with the TIMI score. Table 1. presents the sociodemographic and clinical characteristics of the patients with or without AMI-induced depression.

### 4.3. Interleukin 6 (IL-6) and Depression

**Figure 2:**
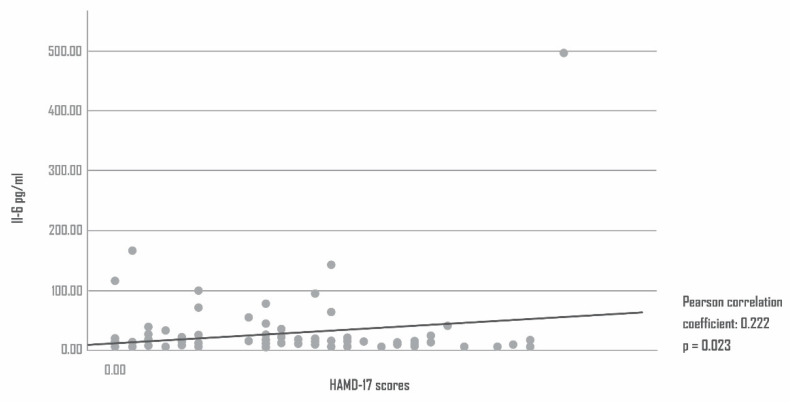
IL-6 levels and HAMD-17 scores.

The IL-6 levels at the time of AMI were significantly positively correlated with the Hamilton rating scale for depression (HAMD-17) scores 6 months after AMI (IL-6 levels in patients with depression 6 months after AMI: mean = 35.77 pg/mL; SD = ± 79.60; IL-6 levels in patients without depression 6 months after AMI: mean = 18.20 pg/mL; SD = ± 22.80; Pearson correlation coefficient: 0.222; *p* = 0.023). By contrast, the Il-6 levels at the time of AMI did not correlate significantly with the HAMD-17 levels at the time of admission (Pearson correlation coefficient: 0.142; *p* = 0.151). Figure 2. shows the IL-6 levels and HAMD-17 scores.

### 4.4. MPO and AMI

In the whole group, the patients had significantly higher MPO concentrations immediately after AMI compared with MPO concentrations 6 months after AMI (MPO at the time of the AMI: 482.75 ng/mL (SD ± 184.53); MPO 6 months after AMI: 398.52 ng/mL (SD ± 179.60); t = −4.676, df = 108, *p* = < 0.001).

### 4.5. MPO and Type of AMI

The study results showed no relationship between MPO concentrations and type of AMI (STEMI, NSTEMI): t = −0.187, df = 107, *p* = 0.852.

### 4.6. MPO and Severity of AMI

The maximum creatinine kinase (CK max) within the first days after AMI is a biomarker for the severity of the AMI. The CK max within the first three days after AMI did not correlate with MPO immediately after the AMI (Spearman’s Rho: 0.092; *p* = 0.343). Furthermore, the creatine kinase MB (CK-MB) and troponin T concentrations did not correlate with MPO immediately after the AMI (CK-MB: Spearman’s Rho: 0.033; *p* = 0.768; troponin T: Spearman’s Rho: 0.007; *p* = 0.940).

### 4.7. MPO and Depression

**Figure 3:**
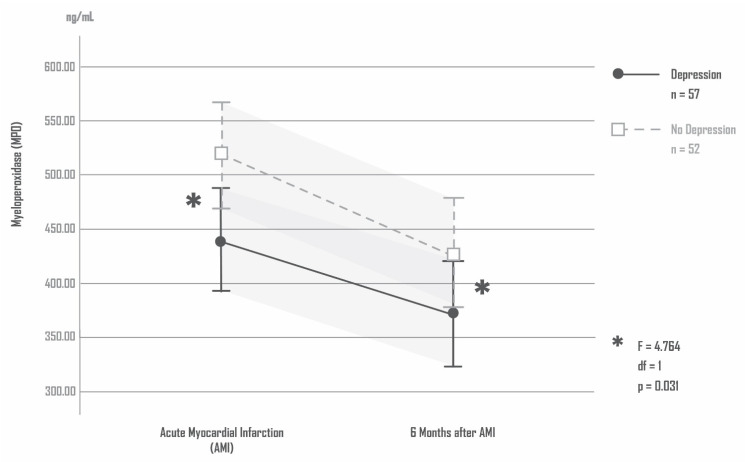
Levels of MPO in patients with and without AMI-induced depression.

The patients with AMI-induced depression 6 months after AMI showed significantly lower MPO blood levels compared to AMI patients without depression immediately and 6 months after the AMI. ANCOVA: MPO (depression) F = 4.764, df = 1, *p* = 0.031; MPO (time) F = 0.309, df = 1, *p* = 0.580; MPO (depression × time) F = 0.466, df = 1, *p* = 0.497; covariate age: F = 0.048, df = 1, *p* = 0.828; covariate sex: F =0.657, df = 1, *p* = 0.419; covariate coronary artery disease (number of affected vessels): F =0.034, df = 1, *p* = 0.854; covariate maximum creatinine kinase (CK max): F =0.206, df = 1, *p* = 0.651; covariate BMI: F =0.330, df = 1, *p* = 0.567. Figure 3. Shows the levels of MPO in patients with and without AMI-induced depression.

### 4.8. MPO and Severity of Depression

**Figure 4:**
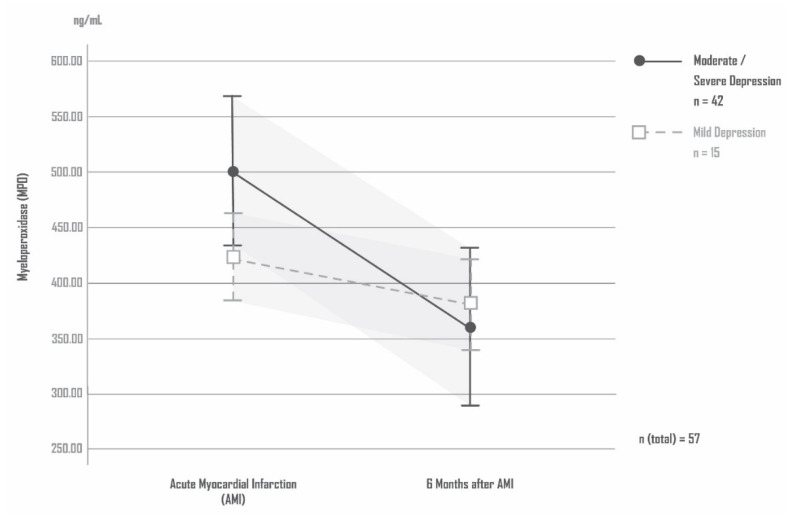
Levels of MPO in patients with mild and with moderate/severe AMI-induced depression.

The patients with AMI-induced moderate or severe depression did not differ significantly in their MPO blood levels from the patients with AMI-induced mild depression. ANCOVA: MPO (time) F = 1269, df = 1, *p* = 0. 265; MPO (depression severity) F = 0.769, df = 1, *p* = 0.385; MPO (depression severity × time) F = 3.828, df = 1, *p* = 0.056; covariate age: F = 2205, df = 1, *p* = 0.144; covariate sex: F =3.937, df = 1, *p* = 0.053; covariate coronary artery disease (number of affected vessels): F =2.764, df = 1, *p* = 0.102. Figure 4 shows the levels of MPO in patients with mild and with moderate/severe AMI-induced depression

### 4.9. Multivariate Analyses

A two-step hierarchical logistic regression model was used to examine the potential correlates of AMI-induced depression. In the first step, we regressed the MPO at the time of hospital admission against AMI-induced depression 6 months after AMI (depression six months after AMI: yes/no) (Model 1). In the next step, we introduced the common control variables age, sex and coronary artery disease (number of affected vessels) into the hierarchical linear regression model (Model 2).

Regarding Model 1, the baseline MPO was a significant predictor of AMI-induced depression (MPO: *p* = 0.027). When the control variables (Model 2) were included in the hierarchical logistic-regression analysis, MPO remained a significant predictor of AMI-induced depression (MPO: *p*= 0.025). Table 2. presents the two-step hierarchical logistic regression models of AMI-induced depression.

By contrast, the MPO 6 months after the AMI is not a significant predictor of AMI-induced depression 6 months after AMI. MPO 6 months after the AMI: B = −0.002, S.E. = 0.01; WALD = 2.030, df = 1, Exp (B) = 0.998, *p* = 0.154.

## 5. Discussion

Many previous studies have shown that AMI patients suffer from severe and prolonged stress [35]. Prolonged AMI-related stress and the concomitant activation of proinflammatory cytokines make AMI patients more vulnerable to depression [11,22,23,24]. Corresponding with this, in this study, the IL-6 levels at the time of AMI were significantly and positively correlated with the Hamilton rating scale for depression (HAMD-17) scores 6 months after AMI.

There is a large body of evidence suggesting that the immune response might be impaired in patients with depressive disorders. In general, major depression is often associated with systemic immune activation, which includes abnormality in inflammatory markers, immune cell numbers, and antibody titers. However, there is also evidence that major depression might be associated with immunosuppression [39,40,41]. Thus, for a long time, deficits in the innate immune system in people with major depression have been suspected [11].

Neutrophils constitute the most important components of the innate immune system and play a major role in the clearance of pathogens [39]. The antimicrobial activity of neutrophils is mainly based on MPO, located in the azurophil granules. MPO generates numerous reactive oxidants and radicals, which cause oxidative cell damage to proteins, lipids, lipoproteins and DNA [42,43,44]. In a previous study of patients who underwent cardiopulmonary bypass surgery, the MPO levels reflected the leukocyte-activation state induced by this kind of elective surgery [45]. Thus, MPO has been proposed “to mirror the degree of neutrophil activation“ [46] and makes it possible to assess the suppression of the innate immunity. 

The results of the present study indicate that patients with AMI-induced depression have significantly lower levels of MPO immediately and 6 months after AMI. These results might reflect a significant depression-related suppression of the innate immune system in depressive patients after AMI. In addition, the data in this study demonstrate that baseline MPO concentrations, which were measured immediately after the AMI, were significant predictors of AMI-induced depression in the long term. 

In agreement with the results of our study, Duggal et al. [27] showed, in a population without known coronary artery disease, consisting of elderly patients with hip fractures, that the stress load led to depressive episodes, a reduction in neutrophil function and poorer neutrophil-superoxide production. 

In a study with male twins without acute cardiovascular disease, Vaccarino et al. (2008) investigated whether blood MPO levels and other markers of inflammation are associated with major depression. The dizygotic twins with major depression had significantly higher serum levels of MPO than those without depression [47]. Furthermore, in a study by Liang et al. (2013), patients with coronary artery disease and depression had higher MPO serum concentrations [48]. This indicates that different forms of depression might exist, which might be reflected in different MPO levels. Gaecki et al. (2010) investigated whether a functional polymorphism of the MPO gene (G-463 A) exists in depressed patients. They compared patients with recurrent depressive disorder and healthy controls, and the results of their study showed differences in genotype distribution and allele frequency between patients with depression and healthy controls [49].

This study did not only reveal the impact of MPO as a potential biomarker of depression caused by an AMI. Considering the total group, regardless of depressive symptoms, the patients had significantly higher MPO concentrations immediately after AMI than at 6 months after AMI. The impact of MPO on the early diagnosis of AMI was evident in a previous study by Oran et al. [50]. 

## 6. Limitations

A comparison of MPO serum levels with those of a control group of healthy individuals should be considered for subsequent studies.

## 7. Conclusions

In this study, MPO was significantly associated with AMI-induced depression. Decreased MPO blood concentrations immediately and 6 months after AMI may reflect a suppression of the innate immune system in depression-vulnerable AMI patients. Thus, MPO might be a suitable biomarker for AMI-induced depression, indicating a depression-associated suppression of the innate immune system.

## Figures and Tables

**Table 1:** Sociodemographic and clinical characteristics of the patients with or without AMI-induced depression.

Category		Total Sample (n = 109)	*p*	Depression (n = 57/109; 52.3%)	No Depression (n = 52/109; 47.7%)	*p*
Sociodemographic Characteristics						
Age	mean (±SD)	60.3 (±11.33)	-	59.84 (±12.50)	60.84 (±9.99)	t = 0.460, df = 107 *p* = 0.646 ^a^
Sex						
Male	n (%)	90 (82.6%)	χ² = 46.248, df = 1, *p* < 0.001 ^b^	42 (73.7%)	48 (92.3%)	χ² = 6.553, df = 1 *p* = 0.010 ^b^
Female	n (%)	19 (17.4%)		15 (26.3%)	4 (7.7)	
Clinical Characteristics						
Type of Acute Myocardial Infarction						
NSTEMI	n (%)	44 (40.4%)	χ² = 2.440, df = 2, *p* = 0.295 ^b^	23 (40.4%)	21 (40.4%)	χ² = 0.135, df = 2, *p* = 0.935 ^b^
STEMI (anterior)	n (%)	33 (30.3%)		18 (31.6%)	15 (28.84%)	
STEMI (posterior)	n (%)	32 (29.4%)		16 (28.1%)	16 (30.8%)	
Killip Class Stratification						
Killip Class I	n (%)	85 (78.0%)	χ² = 199.71, df = 3, *p* < 0.001 ^b^	43 (82.7%)	42 (91.3%)	χ² = 2.798, df = 3, *p* = 0.42 ^b^
Killip Class II	n (%)	7 (6.4%)		4 (7.7%)	3 (6.5%)	
Killip Class III	n (%)	4 (3.7%)		3 (5.8%)	1 (2.2%)	
Killip Class IV	n (%)	2 (1.8)		2 (3.8%)	0 (0%)	
Severity of the AMI						
Maximum Creatinine Kinase (CK max)	Median, IQR	314 U/L, 714.0	-	376 U/L, 732.5	243 U/L, 643.0	Mann-Whitney-U= 1321.0, *p* = 0.329 ^d^
Creatine Kinase MB (CK-MB)	Median, IQR	32.5 U/L, 69.5	-	43.0 U/L, 88.0	30.0 U/L, 62.0	Mann-Whitney-U= 702.5, *p* = 0.109 ^d^
Troponin T	Median, IQR	784 pg/mL, 2153	-	849.0 pg/mL, 2546.0	725.5 pg/mL, 2133.25	Mann-Whitney-U= 1375.0, *p* = 0.518 ^d^
Percutaneous Coronary Intervention (PCI)-Related Parameters						
Coronary Flow before PCI (TIMI Score ^e^)						
0-I ^e^	n (%)	82 (75.2%)	χ² = 107.540, df = 2 *p* < 0.001 ^b^	40 (78.4%)	42 (85.7%)	χ² = 1.886, df = 2 *p* = 0.389 ^b^
II ^e^	n (%)	13 (11.9%)		7 (13.7%)	6 (12.2%)	
III ^e^	n (%)	5 (4.6%)		4 (7.8%)	1 (2.0%	
Coronary Flow after PCI (TIMI Score ^e^)						
0-I ^e^	n (%)	4 (3.7%)	χ² = 151.264, df = 2 *p* < 0.001 ^b^	0 (0%)	4 (7.7%)	χ² = 10.977, df = 2 *p* = 0.004 ^b^
II ^e^	n (%)	7 (6.4%)		7 (13.0%)	0 (0%)	
III ^e^	n (%)	95 (87.2%)		47 (87.0%)	48 (92.3%)	
Multivessel PCI	n (%)	23 (22.5%)	-	14 (25.9%)	9 (18.8%)	χ² = 0.749, df = 1 *p* = 0.387 ^b^
Coronary Artery Disease—Number of Affected Vessels	Mean (±SD)	1.85 (±0.82)		1.89 (±0.86)	1.81 (±0.77)	t = −0.555, df = 107 *p* = 0.580 ^a^
In-Hospital Outcome						
Severe Bleeding	n (%)	0 (0%)	-	0 (0%)	0 (0%)	-
Reinfarction	n (%)	2 (1.8%)	-	0 (0%)	2 (3.8%)	*p* = 0.234 ^c^
LVEF (%)	Mean (SD)	53.05 (±10.893)	-	53.78 (±11.96)	52.21 (±9.59)	t = −0.615, df = 72 *p* = 0.541 ^c^
Cardiological Risk Factors						
Body Mass Index	Mean (±SD)	28.21 (±3.87)	-	27.93 (±4.03)	28.51 (±3.70)	t = 0.752, df = 100, *p* = 0.454 ^a^
IDDM	n (%)	2 (1.8%)	-	2 (3.5%)	0 (0%)	*p* = 0.496 ^c^
Arterial Hypertension	n (%)	98 (89.9%)	-	49 (94.2.7%)	49 (86.0%)	χ² = 2.048, df = 1 *p* = 0.152 ^b^
Hyperlipidemia	n (%)	55 (50.5%)	-	33 (57.9%)	22 (44.9%)	χ² = 1.783, df = 1 *p* = 0.182 ^b^
Previous Mental Illness						
Previous Mental Illness (Adjustment Disorder, Burn-out Syndrome)—not present at the time of the AMI	n (%)	10 (9.2%)	-	8 (14.0%)	2 (3.8%)	*p* = 0.097 ^c^
Addiction to Alcohol	n (%)	1 (0.9%)	-	1 (1.8%)	0 (0%)	*p* = 0.388 ^c^
Addiction to Nicotine	n (%)	50 (45.9%)	-	26 (45.6%)	24 (46.2%)	χ² = 0.003, df = 1 *p* = 0.955 ^b^
Addiction to Illicit Drugs	n (%)	0 (0%)	-	0 (0%)	0 (0%)	-

Legend: ^a^ t-test for independent samples (depression/no depression); ^b^ χ²-test, ^c^ Fisher exact; ^d^ Mann–Whitney-U test, ^e^ TIMI Score: 0 (total occlusion, no perfusion)—3 (normal epicardial perfusion, normal flow); Ck-MB: creatine kinase myocardial band; IDDM = insulin-dependent diabetes mellitus; LVEF = left-ventricular ejection fraction.

**Table 2:** Two-step hierarchical logistic regression models of AMI-induced depression.

Step 1 (Model 1) DV = Depression (Yes/No)	B	S.E.	WALD	df	Exp (B)	*p*
Constant	1.404	0.617	5.181	1	4.073	0.023
MPO at the time of AMI	−0.003	0.001	4.922	1	0.997	0.027
	R^2^ _COX &Snell)_: 0.051 Omnibus: χ² = 5.728, df = 1, *p* = 0.017 Hosmer–Lemeshow Test: χ² = 9.607, df = 8, *p* = 0.294					
**Step 2 (Model 2)** **DV = Depression (Yes/No)**	**B**	**S.E.**	**WALD**	**df**	**Exp (B)**	***p***
Constant	3.759	1.614	5.424	1	42.926	0.020
MPO at the time of AMI	−0.003	0.001	5.013	1	0.997	0.025
Sex (male = 1)	−1.440	0.621	5.372	1	0.237	0.020
Age	−0.026	0.020	1.675	1	0.974	0.196
Coronary artery disease (number of affected vessels)	0.322	0.265	4.646	1	1.380	0.224
	R^2^ _COX &Snell)_: 0.122 Omnibus: χ² = 14.165, df = 4, *p* = 0.007 Hosmer-Lemeshow-Test: χ² = 8.872, df = 8, *p* = 0.795)					

Legend: MPO = myeloperoxidase; AMI = acute myocardial infarction.

## Data Availability

Data are contained within the article and Supplementary Material.

## Supplementary Materials

The following supporting information can be downloaded at: https://www.mdpi.com/article/10.3390/antiox11112083/s1, Data set.
